# COMPLAINT PATTERNS IN US NURSING HOMES: 2013–2017

**DOI:** 10.1093/geroni/igac059.2370

**Published:** 2022-12-20

**Authors:** Kallol Kumar Bhattacharyya, Victor Molinari

**Affiliations:** University of South Florida, Tampa, Florida, United States; University of South Florida, Tampa, Florida, United States

## Abstract

Nursing home (NH) quality of care is often short of meeting residents' and family expectations to maintain optimum quality of life. Using complaints as a facility-level outcome (i.e., complaints per NH), this study updates earlier published findings by replicating prior analyses with more recent data, and by analyzing the number of complaints, complaint allegations, and deficiency citations separately. This will allow us to determine whether any major change has taken place in the consumer complaint pattern in recent years. The result reveals, in the entire study period (2013-2017), overall, 458,101 complaints (5.9/NH/year) were identified that contain 949,466 allegations (12.2/NH/year), which resulted in the issuance of 156,135 deficiency citations (2.0/NH/year) in about 15,600 NHs across the country. Regarding the number of complaints, substantiated complaints, and deficiency citations, the results show a steady increase compared to previous years. Furthermore, there are marked differences among the ten CMS survey regions on the prevalence of overall complaints, substantiated complaints, and deficiency citations. The current study found a lower number of NHs with zero complaints and a higher number of NHs with five/more complaints in later years suggesting a steady increase in the number of complaints over the years. However, the average rate of substantiation of complaint allegations is showing a decreasing trend in recent years. This may be because people are now complaining more due to higher care expectations. Alternatively, it may be simply be because of the easier complaint lodging process developed in recent years. Other policy and practice implications will be discussed.

